# Interventions to reduce dependency in bathing in community dwelling older adults: a systematic review

**DOI:** 10.1186/s13643-017-0586-4

**Published:** 2017-10-11

**Authors:** Miriam Golding-Day, Phillip Whitehead, Kathryn Radford, Marion Walker

**Affiliations:** School of Medicine, The University of Nottingham, Queens Medical Centre, Nottingham, NG2 7UH UK

**Keywords:** Older adults, Bathing disability, Activities of daily living, Quality of life

## Abstract

**Background:**

The onset of bathing disability for older adults has been found to be an indicator and potential precursor of further disability. Thus interventions targeting bathing may prevent or delay further disability and the use of health and social care services. The aim of this systematic review was to identify interventions targeted at reducing dependency in bathing for community dwelling older adults, and determine their content and effectiveness in maintaining or improving function and quality of life.

**Methods:**

We conducted a systematic search of electronic databases including: The Cochrane Central Register of Controlled Trials (CENTRAL); MEDLINE; EMBASE; AMED; CINAHL; PsycINFO and OTSeeker. The search took place on 18 October 2016. We included randomised controlled trials, nonrandomised controlled trials, and controlled before and after studies that evaluated an intervention designed to reduce dependency in bathing. Articles were screened for inclusion by two independent reviewers; risk of bias was assessed using quality assessment tools; and data extracted using pre-prepared forms. Disagreements were resolved by discussion and inclusion of a third reviewer.

**Results:**

The search process identified one study for inclusion in the review. This study evaluated a bathing intervention delivered by an occupational therapist following discharge from hospital. Overall, the findings suggest modest improvements in functional ability in favour of the intervention group although the results should be interpreted with caution.

**Conclusion:**

Despite evidence suggesting the importance of addressing bathing difficulties as a means of possible prevention of disability in the ageing process, there is a dearth of evaluative or interventional research studies. Further robust research is warranted, including studies of randomised and controlled design.

## Background

The relationship between ageing and limitations in functional ability is becoming ever more recognised [1], with an increasing awareness of the impact of factors affecting an individual’s ability to ‘age well’ [2, 3]. With increased prevalence of aged people, the international community is placing increasing aegis on identifying and evidencing interventions which might reduce functional and societal disability for older adults. Furthermore, the Care Act 2014 has placed a responsibility on local authorities in England to provide services which prevent or delay the need for care and support [4]. The onset of bathing disability has been shown to be a seminal point in the disabling process for older adults [5, 6]; it may, therefore, be a judicious time point for intervention to prevent the onset of further disability, and reduce the need for health and social care services.

A cohort study conducted in the USA followed 754 non-disabled adults, aged over 70 years, every month for 6 years with regard to their difficulties in performing activities of daily living (ADL) [5]. Over the course of the study, those who developed a disability in bathing were five times more likely to develop a disability in another activity of daily living the following month. This suggests that developing a bathing disability may be an indicator and potential precursor of further disability. Gill et al., [5] concluded that programmes designed to restore and maintain independent bathing for older adults have the capacity to prevent further deterioration in functional ability. It is possible that such programmes may have a preventative effect on the disabling process.

The purpose of this systematic review was to determine whether interventions targeted at bathing could reduce dependency in other activities of daily living (ADL). The focus was to identify studies that compared an intervention designed to reduce dependency in bathing for older persons living in the community, with provision of routine care where there was no explicit intention to actively address bathing disability. In focussing on studies of evaluative design, our aim was to determine whether there was evidence that bathing interventions could prevent the onset of further disability and, consistent with the principles of the Care Act 2014 [4], the need for additional health and social care support.

There were three objectives for this review:

1. To determine what interventions targeting the reduction of dependency in bathing for older adults have been provided and evaluated in the literature.
2. To determine the efficacy and effectiveness of these interventions on older adults’ dependency in bathing, functional (ADL) ability and quality of life.
3. To determine whether bathing interventions prevent the need for, and use of, other health and social care services.

## Methods

The review was registered in the PROSPERO database (CRD42016048818) [7] and conforms to the PRISMA statement [8]. Randomised controlled trials (RCTs), non-randomised controlled trials, controlled before and after studies and interrupted time series were all eligible for inclusion. Participants included individuals aged 60 years and older, living at home in the community (i.e. not in a residential or nursing care home) and who had been assessed as having a bathing disability. For this review, we did not restrict by diagnosis but required participants to have had a professional assessment which identified difficulty in bathing/a bathing disability. We defined bathing disability as being unable to use, or having difficulties in using, existing bathing facilities in the home environment. Studies where otherwise healthy participants acquired a perceived disability through artificial environmental changes were excluded.

To be included in the review, interventions had to be bathing specific and targeted at reducing dependency in bathing for older adults. Interventions could take the form of home modification/adaptations to aid the bathing process or be in the form of input from a health or social care professional to modify the task or activity, or to enable participants to better use equipment and bathing devices in order to bathe independently. Studies with a control group were eligible for inclusion where standard care was used as the control, or where persons would receive normal packages of care or intervention in line with local practice.

The main outcome of interest was performance in personal ADL with specific focus on washing, bathing/showering but to also include dressing, feeding and toileting, management of continence, transfers and basic mobility. An outcome could take the form of an activity of daily living score such as the Barthel Index or self-reported difficulties in ADL. Other outcomes included: dependence in bathing; perceived difficulty in bathing; health-related quality of life (QoL); social care-related QoL; service use outcomes; death; performance in extended ADL (for example shopping, outdoor mobility); admission/number of admissions to hospital, residential or nursing care homes; participant mood/morale; falls; health economic outcomes; caregiver strain/burden; participant and carer satisfaction with services and healthcare provider satisfaction with services.

The following databases were searched for studies prior to October 2016: the Cochrane Central Register of Controlled Trials (CENTRAL), MEDLINE (1948 to present), EMBASE (1980 to present), AMED (1985 to present), CINAHL (1982 to present), PsycINFO (1967 to present) and Occupational Therapy database for systematic reviews and randomised controlled trials (OTSeeker; 1980 to present). The search was conducted in English and the strategy consisted of a combination of subject headings and free text terms. The search strategy for MEDLINE is shown in Table 1, this was adapted for use in the other databases.

**Table 1:** MEDLINE systematic review search strategy

1. older adults/	
2. elderly/	
3. aged/	
4. (older$ adj1 (person$ or adult$)).tw.	
5. (elderly$ adj2 (adult$ or person$)).tw.	
6. 1 or 2 or 3 or 4 or 5	
7. baths/	
8. shower$.tw.	
9. (bath$ adj3 (independ$ or depend$ or disab$)).tw.	
10. 7 or 8 or 9	
11. 6 and 10	
12. randomized controlled trial.pt.	
13. controlled clinical trial.pt.	
14. (control$ adj2 trial).tw.	
15. intervention study/	
16. experiment$.tw.	
17. (time adj1 series).tw.	
18. (pre test or pretest or posttest or post test).tw.	
19. random allocation/	
20. intervention?.tw.	
21. evaluation studies/	
22. comparative study.pt.	
23. 12 or 13 or 14 or 15 or 16 or 17 or 18 or 19 or 20 or 21 or 22	
24. 11 and 23	
25. nursing home/	
26. 24 not 25	
27. radon/	
28. 26 not 27	
29. limit to adults	

Reviewers followed a three-stage screening process where, firstly, two reviewers (MG-D, PW) examined the titles and excluded studies not pertinent to the review. Secondly, the abstracts of all remaining records were screened independently by both reviewers. Finally, full-text copies of those deemed potentially relevant were screened independently by both reviewers for inclusion. Data was then extracted using pre-prepared and piloted data extraction forms, independently and in duplicate, by two reviewers (MG-D, PW). The extracted information included study methodology, study setting, study population and participant demographics and baseline characteristics, details of the intervention and control, recruitments and drop-out rates and outcome measurements and timing. The results of this data extraction were compared and discussed; any disagreements resolved in consultation with a third reviewer (KR).

Two reviewers (MG-D, PW) also used the quality assessment criteria developed by the Cochrane Effective Practice and Organisation of Care (EPOC) review group [9], to independently assess the methodological quality of the included studies and to identify any risk of bias. This covered sequence generation and allocation concealment, baseline characteristics and measurements, completeness of outcome data, blinding of outcome assessment and selective outcome reporting, as well as other potential sources of bias. Each of these characteristics were explicitly rated and categorised as being of low, high or uncertain risk of bias, and any disagreement about which rating was most suitable was resolved by discussion with a third reviewer (KR).

## Results

**Fig. 1:**
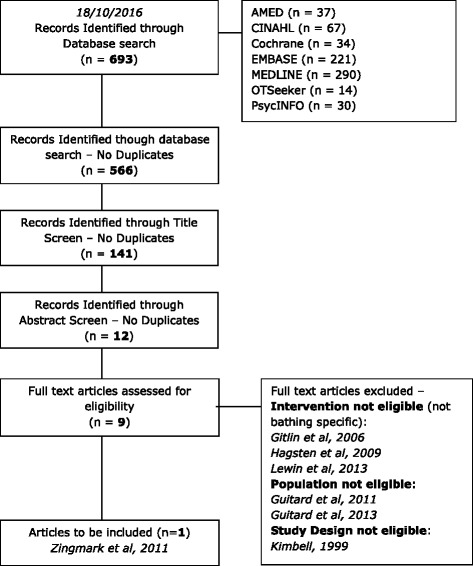
Flow diagram of search process

The search process is summarised in Fig. 1. The reviewers identified 693 records through electronic database searches, with 566 original works once duplicates were removed. One hundred forty-one records were included following a title screen, with nine remaining once abstracts were assessed for suitability. Out of those nine, one was deemed suitable for inclusion after the full text had been obtained and read by both reviewers. A summary of study characteristics is given in Table 2, and the findings of the risk of bias assessment [9] are shown in Table 3. The final study for inclusion in this review was found to be of high risk in five domains, indicating an overall high risk of bias.

**Table 2:** Characteristics of included study

Country of study and reference	Study type	Participants	Primary outcome	Follow-up months
Sweden, Zingmark and Bernspang [10]	CBA	74 adults aged > 65 years, in process of applying for help with bathing.	Ability to perform ADL (ADL taxonomy)	4 (15 weeks)

**Table 3:** Risk of bias summary

Study	A	B	C	D	E	F	G	H	I
Zingmark and Bernspang [10]	H^a^	H^a^	H	L	H	H	L	L	L

*H* high risk, *L* low risk; *U*, unclear risk

^a^Automatically rated due to study type

The included study [10] focussed on the provision of home care services in northern Sweden. Participants were not randomised, but divided into treatment and control groups due to locality. One area acted as the control providing usual care, the second provided an occupational therapist visit and assessment following hospital discharge. They then delivered interventions in accordance with the client’s individual needs in an attempt to improve their ability to bathe independently. These interventions took the form of an encouraging approach as well as a gradual withdrawal of support as appropriate for each client. Sixty percent of clients also received assistive devices and/or environmental adaptions to aid with bathing activity. The authors presented their findings graphically, with measures based on the client’s perceived difficulty in performing each task rather than their actual task completion. The primary domain in the ADL taxonomy scale associated with bathing was classified as ‘washing body’, with two others that can be considered supplementary including ‘washing hair’ and ‘washing hands and face’. Significant improvements were reported in both groups for ‘Washing body’, whilst only those in the intervention group reported significant improvement in ‘Washing hair’.

As well as evidencing an individual’s ability to perform ADLs, secondary outcomes were also recorded by the authors. The EQ-5D questionnaire was used to assess health-related quality of life. This was also presented graphically with both groups improving significantly in ‘self-care’ and ‘usual activities’, and a further two, ‘mobility’ and ‘pain/discomfort’, demonstrated significant improvement only in the intervention group. Overall clients in both groups demonstrated an improved health-related quality of life in total. The study also provided additional outcomes in the form of number of homecare support hours, death of client and number of admissions to hospital, residential or nursing care. Of particular interest was the recorded effect on allocation of home help, with only 30% of those in the intervention group being allocated home help in comparison to 75% in the control group. The average weekly time given for home help with bathing was also significantly decreased, with those in the intervention group only requiring 31 min, and those in the control group averaging 66 min. These differences suggest the intervention has an effect of reducing the citizens need for home help, though we must consider the discrepancy may also be due to local differences in service delivery or availability of support between the two municipalities.

The search, though comprehensive, only returned one study, which was limited by its small sample size, non-random allocation of intervention and some differences between groups at baseline. This was compounded by the use of a non-standardised ADL measure, which limits the primary outcomes comparability with other studies. However, the outcomes of this study tentatively suggest that occupational therapist, specific targeting of a bathing disability, might decrease the need for additional social care involvement and increase overall health-related quality of life.

## Discussion

Despite observational studies indicating that the onset of bathing disability is an important event in the disabling process for older adults, we found a dearth of evidence from interventional studies on bathing using evaluative study designs. Most noticeable was the distinct lack of randomised or controlled trial methodologies. The search did, however, identify one study providing tentative evidence to support the use of interventions to reduce bathing disability in older adults living in the community. In adopting a focussed scope for this review, which included a specific population group, specific intervention and specific study designs, the intention was to identify evaluative studies with strong, replicable results. However, very few studies were identified as being suitable for analysis. Due to the return of only one study, a meta-analysis was not possible. The very limited number of studies which were eligible for inclusion in the review highlights the specialist nature of this research field and the restricted evidence available currently.

We identified two other potentially relevant studies, which partially met the inclusion criteria, but which were excluded on the grounds of population age. Chamberlain et al., [11] conducted an evaluation of bathing aids and equipment by accelerating provision following discharge from hospital compared to usual care. However, they did not limit the age of the population to those over 60 and thus were excluded from the analysis. Chiu and Man [12] evaluated whether additional training in the use of assistive bathing devices led to a higher level of functioning and independence for older adults following a stroke. Participants for this study were aged between 55 and 92 years old, and thus this study was also excluded as being outside our pre-determined age range. The results of both studies suggested that the intervention groups had better outcomes in terms of independence in bathing [11] and better functional independence [12]. However, both were based on small sample sizes.

It is possible that other studies may have been identified for inclusion if we had included a wider age range than the specified ≥ 60 years. However, the rationale for this review was based on evidence gathered from observational cohort studies specifically with older adults [5], indicating the particular importance of bathing for this population. This highlights the need for further robust research in this area, with the older adult population specifically.

We believe the search strategy used in this review was extensive and comprehensive; however, there is a possibility some studies were not identified during the search process. In particular, the need to adapt key words and terms for the different electronic databases meant that some studies may have been missed, and it is possible that all relevant studies were not identified. Additionally, all the searches were conducted in English and so studies without an English abstract were not included. Furthermore, search terms for non-randomised studies are less well developed and there is a possibility that some studies may not have been identified.

We searched only for controlled trials or interrupted time series analyses because our aim was to evaluate the potential preventative effect of bathing interventions. This strict study design criterion was a limiting factor in the amount of studies eligible for inclusion. The search showed that research across all designs is significantly limited in this topic area, particularly evaluative designs. Evaluative designs were of particular interest to the reviewers as with the introduction of the Care Act 2014 [4], there is an increased emphasis on providing evidence for prevention interventions that has parity with other health and social care interventions. Conducting and collating such controlled trials are, therefore, of significant importance within current social care provision and the prevention agenda. However, qualitative studies have also been conducted into bathing interventions and we would recommend a further complementary synthesis of qualitative works to add to the understanding of the mechanisms of the potential preventative effects of bathing.

To our knowledge, this is the only systematic review to date which has collated studies related to bathing. The result of the single study included in the review suggests that interventions such as providing bathing aids, adopting an encouraging approach and the installation of adaptations may potentially reduce dependency in bathing for this population group. However, the authors suggest that further research is needed, recommending randomisation and masked evaluation for future studies. We believe the results of the review highlight this need, and also advocate for further research which seeks to determine the relationship between bathing disability and its effect on the older populations, so that the mechanism can be better understood and thus targeted by timely and effective interventions. The authors believe this review would be of most interest to those working both at a personal and policy level within the prevention agenda for older adults. Principally, the review has emphasised the need for evaluative study designs to further investigate the findings from observational studies on the onset of bathing disability and the subsequent decline in ability to perform other ADL. Currently, we are aware of one ongoing study examining the effect of bathing adaptations as an intervention for older people living in the community [13]; however, this study is yet to report its results.

## Conclusion

A key consideration for further studies should be the use of standardised ADL outcome measures and scales to ensure comparability between studies and facilitate future evidence synthesis. Further research in this area should also explore the wider and longer-term effect bathing specific interventions have for older adults, as well as the potential impact for population groups of different ages. For future research, we advocate for study designs which incorporate randomisation of participants, adding quantitative authority to the current evidence available.

The identification of only one relevant study is an important finding demonstrating the limited evidence in this area. Given the focus on prevention and delaying the disabling process in older adults in current national and international policy, the lack of evidence is surprising. As bathing has been identified as an important precursor to the onset of difficulty in other ADL, the limited number of studies evaluating whether bathing interventions do prevent, or delay, the onset of further disability is a particular paradox. Further research is required with specific focus on interventions designed to reduce dependency in bathing which are yet to be evaluated.
